# An Annealing Strategy for Inhibiting Recrystallization in Nickel-Based Single-Crystal Superalloys

**DOI:** 10.3390/ma18143341

**Published:** 2025-07-16

**Authors:** Xing Hu, Fuze Xu, Menglin Gao, Shengjun Xia, Shuaiheng Liang, Chunfa Huang, Dexin Ma, Qiulin Li

**Affiliations:** 1 Institute of Materials Research, Shenzhen International Graduate School, Tsinghua University, Shenzhen 518055, China; hu-x22@mails.tsinghua.edu.cn (X.H.);; 2 School of Mechanical and Electrical Engineering, Central South University, Changsha 410083, China; 3 Shenzhen Wedge Central South Research Institute Co., Ltd., Shenzhen 518045, China; 4 Powder Metallurgy Research Institute, Central South University, Changsha 410083, China

**Keywords:** single-crystal superalloy, annealing, recrystallization, microstructure

## Abstract

The inhibition of recrystallization in high-strain nickel-based single-crystal superalloys remains a critical challenge for advanced turbine blade applications. This study investigates the evolution of the primary γ’ phase and dislocation during annealing in a third-generation Re-containing single-crystal superalloy (WZ30) subjected to 5% compressive deformation. Isochronal annealing (700 to 1200 °C, 1 min) combined with scanning electron microscopy (SEM) and an electron backscatter diffraction (EBSD) analysis revealed a nonlinear variation of the geometrically necessary dislocation (GND) density, which reached a minimum of 1000 °C with 62.7% of the primary γ’ phase retained. Prolonged recovery annealing at 1000 °C for 10 h effectively inhibited recrystallization during subsequent solution heat treatment. This result provides a practical strategy for inhibiting recrystallization in single-crystal superalloys.

## 1. Introduction

Nickel-based single-crystal superalloys are commonly utilized in turbine blades for aircraft engines due to their exceptional mechanical strength at high temperatures, creep resistance, and resistance to fatigue fracture propagation [1]. However, the complex structure of modern single-crystal turbine blades frequently causes localized deformation due to compressive stresses during casting formation and machining operations, which causes recrystallization during solution heat treatment [2,3,4]. This recrystallization generates newly formed high-angle grain boundaries (HABs), significantly reducing the high-temperature endurance lifetime [5,6]. This risk becomes particularly pronounced in higher-generation single-crystal alloys, where conventional recovery annealing processes prove ineffective when strain exceeds 1–2% [7,8,9], severely constraining the service reliability of single-crystal turbine blades.

Recent significant progress has been made in understanding the recrystallization mechanisms of nickel-based single-crystal superalloys. When localized high-energy regions are induced by deformation within the material, subsequent heating to critical temperatures triggers the dissolution of the γ’ phase. This dissolution process readily facilitates the formation of cellular structures in the γ’ phase-dissolved zones [10]. Surface recrystallization in single-crystal alloys predominantly initiates in both dendritic core regions [11,12] and inter-dendritic regions [13,14]. And grinding [15], chemical coatings [16,17], carburization treatments [18,19], recovery heat treatments [20,21,22] were used to inhibit recrystallization. Recovery heat treatment has emerged as the most industrially viable method [23]. Based on the supplementary materials in reference [22], the recovery heat treatment strategies listed in the literature are unable to effectively inhibit recrystallization once the compressive strain reaches 5%. This limitation underscores the need for optimized recovery strategies to better control recrystallization under high compressive strain conditions. In contrast to early generation single-crystal alloys, the recrystallization behavior of third-generation nickel-based single-crystal superalloy (containing 5 wt.% Re) exhibits significant intergenerational disparities. The pronounced grain boundary segregation propensity of elevated Re content intensifies kinetic barriers during recrystallization processes [24], whereas the interaction mechanisms between γ’-strengthening phases (with a volume fraction of approximately 70%) and dislocation configurations remain elusive.

Although existing studies have separately addressed the pinning effects of primary γ’ phases on grain boundary migration [25,26] and thermodynamic prerequisites for recrystallization driven by dislocation-stored energy [27,28], the dynamic synergistic regulation mechanisms between these factors under high-strain conditions (ε > 5%) still lack systematic elucidation. In practice, both the primary γ’ phase and dislocations play crucial roles in recrystallization [13,29]. The primary γ’ phase impedes grain boundary migration, which prevents recrystallization, while dislocations supply the necessary energy for nucleation. Thus, understanding their behavior at various temperatures is key to optimizing recovery annealing processes.

In this study, therefore, the aim is to explore an annealing strategy that inhibits recrystallization in deformed Ni-based single-crystal superalloys. Specifically, the focus is on the evolution of dislocation density and primary γ’ phase content at various temperatures, with the goal of developing an efficient annealing treatment to inhibit recrystallization during solution heat treatment. This research provides a practical solution for addressing recrystallization in high-strain nickel-based single-crystal superalloys.

## 2. Materials and Methods

Cylindrical specimens with dimensions of 10 mm diameter × 15 mm height were extracted from [001]-oriented as-cast rods of the third-generation nickel-based single-crystal superalloy WZ30 (Shenzhen Wedge Central South Research Institute Co., Ltd., Shenzhen, China) produced using the spiral grain selection method in a Bridgman furnace (ALD vacuum precision casting furnace, ALD Vacuum Technologies GmbH, Hanau, Germany). The specimens were oriented in the same direction as the casting direction in the Bridgman furnace to maintain crystallographic alignment. Twelve wax test rods were periodically assembled to form a ceramic shell mold before casting. The mold was then preheated to 1550 ± 5 °C, and the metal melt was poured at 1530 °C, followed by directional solidification at a withdrawal rate of 3 mm/min. The nominal composition of the alloy was Ni-5.8Al-6Co-3.5Cr-0.4Mo-5Re-8Ta-6.5W-0.1Hf (wt.%), calculated from the amounts of alloying elements used in the alloying process. Room-temperature uniaxial compression tests were conducted on a Gleeble 3800 thermomechanical simulator (Data Sciences International, Inc., Plymouth, MN, USA) equipped with a silicon nitride ceramic anvil under a constant nominal strain rate of 1 × 10^−3^ s^−1^, achieving a 5% engineering strain by measuring the height reduction using vernier calipers. The post-deformation specimens were sectioned parallel to the compression axis ([001]) into rectangular blocks (10 × 5 × 1.5 mm^3^) for subsequent heat treatment investigations.

The heat treatments were performed in three stages. To analyze the primary γ’ phase and geometrically necessary dislocations (GND), the samples were rapidly placed in preheated muffle furnaces, stabilized at temperatures ranging from 700 to 1200 °C for 1 min of isothermal holding, followed by air-cooling. The recovery studies involved controlled heating (10 °C/min) to target temperatures of 950 °C, 1000 °C, and 1050 °C in separate experiments, each maintained for 10 h before air-cooling. It should be pointed out that a 10 h response time for the heat treatment is a commonly used response time in factories for low-strain recovery heat treatment. The recrystallization behavior was evaluated through a three-step solution treatment: initial heating from ambient to 1270 °C at 9 °C/min, followed by a slow ramp (0.1 °C/min) to 1340 °C, culminating in a 6 h dwell at 1340 °C, followed by air-cooling. The heating rate parameters were controlled using the KSL-1700X-A2 muffle furnace system (Hefei Kejing Materials Technology Co., Ltd., Hefei, China), while the cooling method followed the standard solution heat treatment protocol established for the WZ30 nickel-based single-crystal superalloy.

The microstructural characterization was performed on the (001)-oriented cross-sections using SEM, EBSD and energy dispersive X-ray spectroscopy (EDS) techniques (Helios 5 UX, Thermo Fisher Scientific Inc., Waltham, MA, USA). Prior to the characterization, the surface preparation included mechanical grinding with progressively finer SiC papers (up to #5000 grit) and final electro-polishing in a methanol-perchloric acid electrolyte (CH_3_OH:10 vol.% HClO_4_) at −30 °C (30 V DC, 70 s). The EBSD mapping was conducted using an Oxford Instruments Symmetry S2 detector (Oxford Instruments, Abingdon, United Kingdom) equipped with AZtecCrystal 2.2 software, employing a 0.5 μm step size for GND and 5 μm for the recrystallization analysis, respectively.

## 3. Results and Discussion

**Figure 1:**
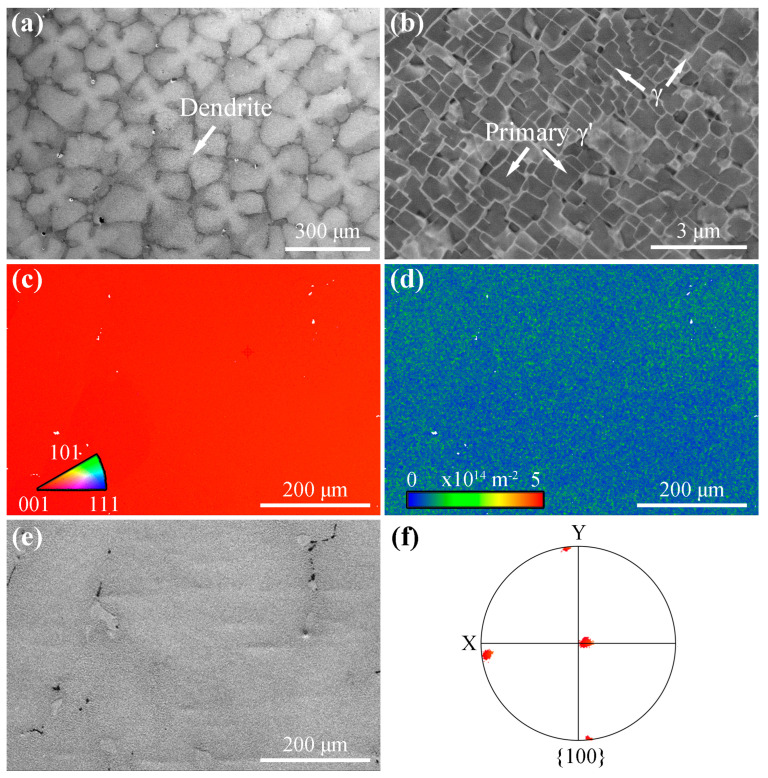
SEM and EBSD maps of the 5% compressed specimens: (**a**,**b**) SEM, (**c**) EBSD IPF maps, (**d**) EBSD GND map corresponding to (**c**), (**e**) EBSD band contrast map corresponding to (**c**), and (**f**) EBSD PF map corresponding to (**c**).

**Figure 2:**
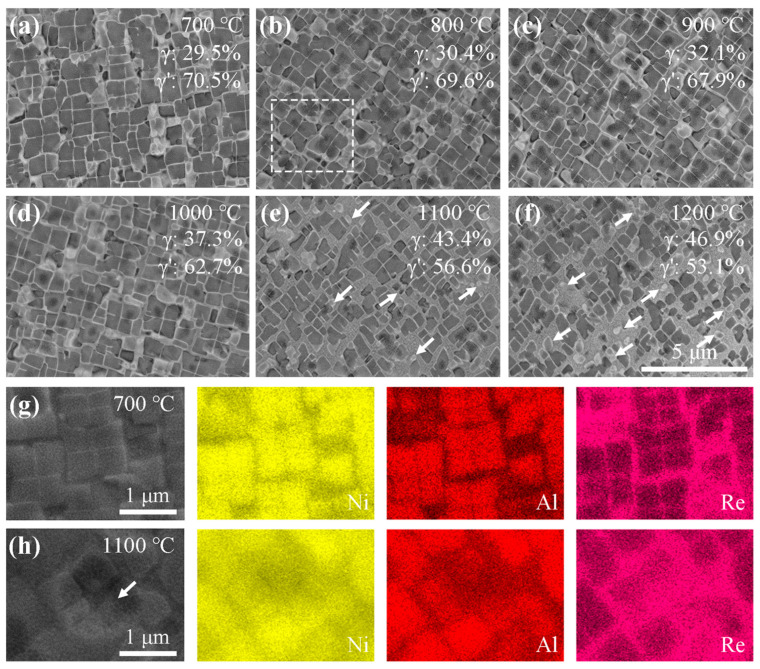
SEM characterization results illustrating microstructural evolution under varied annealing temperatures: (**a**) 700 °C, (**b**) 800 °C, (**c**) 900 °C, (**d**) 1000 °C, (**e**) 1100 °C, and (**f**) 1200 °C. The primary γ’ phase gradually decreases with increasing temperatures, from 70.5% to 53.1%. The ESD characterization results of elemental distribution characteristics in typical microstructures at different temperatures: (**g**) 700 °C and (**h**) 1100 °C. The white dashed box regions highlight the indistinct phase boundaries, and white arrows indicate the dissolving primary γ’ phase. Significant dissolution behavior is observed during the 1100 °C heat treatment, with Ni, Al and Re elements showing segregation.

The SEM and EBSD characterization results of the 5% compressed specimens are presented in Figure 1. Figure 1a displays the dendritic structure known as a ‘cross’ in SEM pictures. Figure 1b illustrates the γ/γ’ morphology, where the primary γ’ phase is evident as square-shaped precipitates dispersed throughout the γ matrix [30]. ImageJ (1.51j8) analysis revealed that the primary γ’ phase accounts for 71.7% of the volume. The inverse pole figure (IPF) map in Figure 1c indicates the absence of grain boundaries or any additional colors representing different orientations. Indications imply that the specimen consists of a single grain that is aligned in parallel with the [001] direction. The GND distribution depicted in Figure 1d demonstrates the plastic deformation with a GND density of 3.8 × 10^13^ m^−2^. The black area in the band contrast map (Figure 2e) corresponds to the shrinkage hole. Figure 1f depicts the pole figure (PF) associated with the grain seen in Figure 1c, illustrating that the crystal is aligned with the Z direction in the {001} PF.

The SEM characterization results of the deformed specimen subjected to 1-min annealing treatments at various temperatures are presented in Figure 2a–f. Upon annealing at 700 °C (Figure 2a), no significant microstructural changes were observed compared with the non-annealed specimen (Figure 1b), with well-defined contours of the primary γ’ phase remaining distinguishable. When the annealing temperature increased to 800 °C, morphological alterations occurred in the primary γ’ precipitates by their progressively indistinct phase boundaries, such as the white dashed box regions in Figure 2b. The subsequent elevation of the annealing temperature to 1100 °C induced the substantial dissolution of the primary γ’ phase, as particularly demonstrated in Figure 2e, which reveals the partial dissolution of these precipitates. Further increasing the temperature to 1200 °C promoted the additional dissolution of the primary γ’ phase, though complete dissolution was not achieved even at this elevated thermal condition. Figure 2g reveals that Ni and Al elements are predominantly distributed within the γ’ phase, while the Re element is primarily concentrated in the γ matrix. Upon annealing at 1100 °C, Figure 2h demonstrates the outward diffusion of Ni and Al from the γ’ phase into the γ matrix, indicating the progressive dissolution of the γ’ phase.

**Figure 3:**
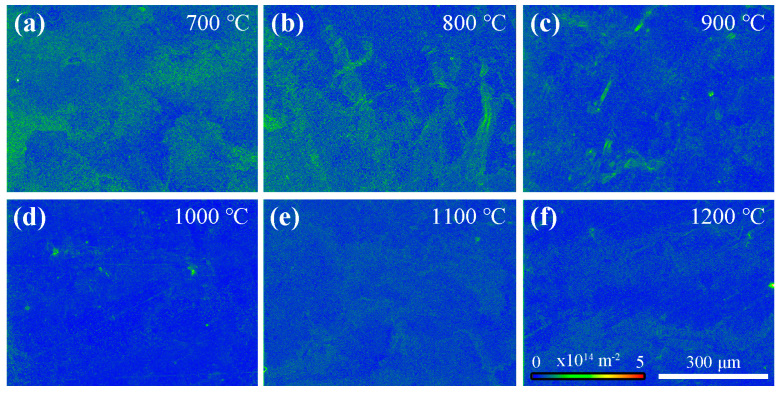
EBSD-GND characterization results of the samples annealed at different temperatures: (**a**–**f**) correspond to annealing temperatures of 700 °C, 800 °C, 900 °C, 1000 °C, 1100 °C, and 1200 °C, respectively. The non-monotonic GND evolution is observed during the heat treatment process. The GND density gradually decreases as the temperature increases up to 1000 °C, but at 1100 °C, it no longer decreases and instead increases.

The EBSD characterization results of samples annealed at different temperatures are presented in Figure 3. The GND density was employed to analyze the dislocation distribution within the specimens. At 700 °C, the initial GND density measured 3.8 × 10^13^ m^−2^ showing minimal variation upon increasing the temperature to 800 °C (3.6 × 10^13^ m^−2^). A marked decline became evident at 900 °C (2.7 × 10^13^ m^−2^), which intensified dramatically at 1000 °C (2.0 × 10^13^ m^−2^). However, after annealing at 1100 °C, the GND density increased to 2.6 × 10^13^ m^−2^. Further elevating the annealing temperature to 1200 °C resulted in a reduction of the GND density compared to the 1100 °C-annealed specimen. This non-monotonic evolution suggests complex thermal activation and recovery mechanisms governing dislocation annihilation/reconfiguration during high-temperature processing.

**Figure 4:**
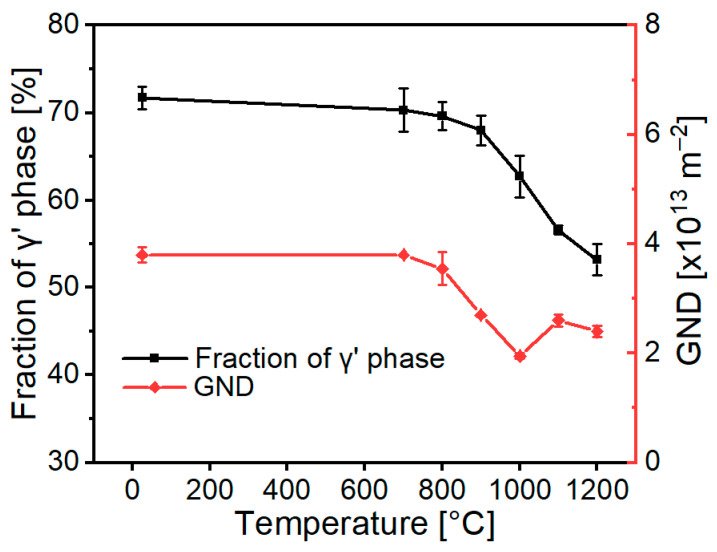
Statistical evaluation of the primary γ’ phase volume fraction and GND density evolution under multi-temperature annealing. The non-monotonic GND evolution is observed at 1000 °C.

To systematically investigate the evolution of the primary γ′ phase distribution and GND density in 5% deformed samples during annealing treatment, five examinations were conducted at each temperature. From Figure 4, it is evident that after annealing at 800 °C, both the primary γ’ phase volume fraction and GND density slowly decreased compared to annealing at 700 °C. As the annealing temperature rises, the primary γ’ phase volume fraction gradually drops from 71.7 ± 1.3% to 53.2 ± 1.8% from room temperature to 1200 °C. However, the GND density does not show a consistent decrease with temperature increases. At 1100 °C, it notably surpasses that at 1000 °C.

Combined with the SEM and EDS characterization in Figure 2, it was revealed that partial dissolution of the primary γ’ phases occurred during the 1100 °C heat treatment accompanied by a reduction in the γ’ phase content (Figure 2e) and the outward diffusion of Al from the γ’ phase into the γ matrix (Figure 2h). The dissolution of the primary γ’ phase promotes the reduction of the GND density [31,32]. The inherent lattice mismatch between the γ matrix and γ’ precipitates (typically δ = 0.1–0.5%) [33,34], though partially relaxed by the dislocation during the early dissolution stage [21,35], the dissolution process still induces local plastic deformation through the following mechanisms: (1) Under the driving force of thermally activated energy at high annealing temperatures, entangled dislocations in the γ-channel rapidly migrate toward the γ/γ′ interface, which leads to an increase in the dislocation density at the γ/γ′ interface, resulting in the formation of an interface dislocation network. However, the formation of this dislocation network hinders further dislocation motion, causing a slight increase in the dislocation density in the observed region [22,36]; (2) At 1100 °C, only a portion of the primary γ′ phase dissolves, and the undissolved primary γ′ phase still exerts a significant pinning effect on dislocations [37,38]. Furthermore, the relatively narrow γ-channel aggravates the accumulation of dislocations after rearrangement.

**Figure 5:**
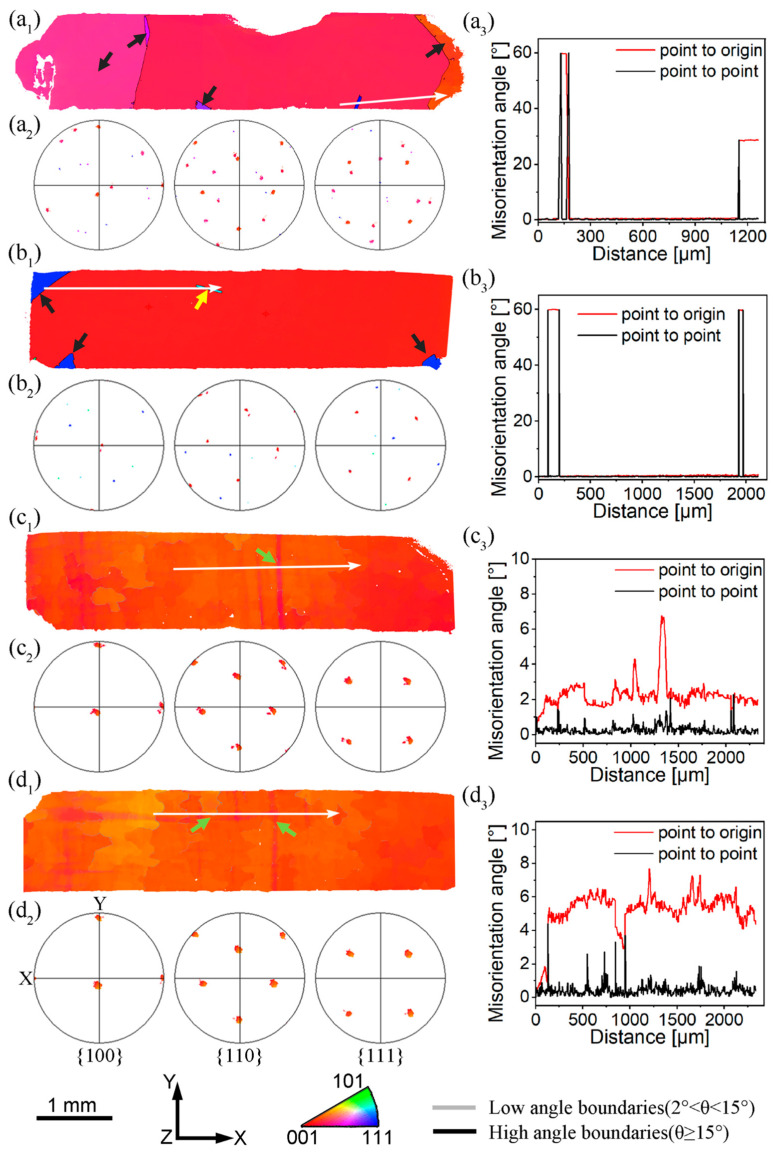
EBSD maps showing the solution heat treatment microstructure of 5% compressed specimens with (**a_1_**–**a_3_**) without annealing, (**b_1_**–**b_3_**) 950 °C–10 h, (**c_1_**–**c_3_**) 1000 °C–10 h, and (**d_1_**–**d_3_**) 1050 °C–10 h annealed, including IPF maps, PF maps, and misorientation distribution maps along the white arrowed path in IPF maps. Black arrows indicate the recrystallized grains. No recrystallized grains were observed after 1000°C–10 h and –15 h recovery heat treatments. Yellow arrows indicate Σ3 twin boundaries, and green arrows indicate regions of local misorientation within the grains, but without the formation of the recrystallized grains, with misorientation angles of less than 10°.

Based on the results in Figure 4, the lowest GND density is observed at an annealing temperature of 1000 °C while still retaining a significant amount of primary γ’. This suggests that 1000 °C may be the optimal temperature for recovery annealing to inhibit recrystallization. Thus, isochronous recovery annealing experiments were conducted on the 5% room-temperature compressed specimens at 950 °C, 1000 °C, and 1050 °C for 10 h, respectively, followed by a solution heat treatment. The results are shown in Figure 5. The solution heat treatment after 950 °C annealing results in internal recrystallization, while annealing at 1000 °C and 1050 °C effectively inhibits recrystallization. Low-angle grain boundaries and lattice distortions are observed within the unrecrystallized specimens, indicating partial grain reorientation.

Through Figure 5(a_1_,b_1_,c_1_,d_1_), it is clear that new orientations were developed during recrystallization nucleation because of the different orientation features in Figure 5(a_1_,b_1_). The nucleation mechanisms for recrystallization have been reported [39,40]; namely, the strain-induced grain boundary migration (SIBM) and orientation-gradient-related subgrain coalescence. The SIBM mechanism typically operates on both sides of the HABs that have varying stored energy. The newly recrystallized grain orientation is primarily inherited from the pre-existing grains, which contrasts with our findings. The second mechanism primarily involves the presence of significant orientation gradients and a high amount of stored energy from the GND, and new orientations will be formed during the formation of recrystallization nuclei. In this study, the compressed single-crystal superalloy was without any HABs. After the solution heat treatment, new grains composed of HABs are formed, exhibiting crystallographic orientations distinct from those of the deformed single crystal. Furthermore, significant orientation gradients were observed within the unrecrystallized specimens in Figure 5(c_3_,d_3_), indicating that even in non-recrystallized samples, subtle crystallographic orientation variations have occurred. Therefore, it can be inferred that the orientation-gradient-related subgrain coalescence mechanism significantly influenced the nucleation process in single-crystal superalloys.

As previously described, the recrystallization in nickel-based single-crystal superalloys is affected by the primary γ’ phases and dislocation density. At lower heat treatment temperatures (approximately 0.5 $T_{m}$ or below, where $T_{m}$ is the melting point), the γ/γ’ microstructure exhibits exceptional stability due to the high concentration of refractory elements (Ni, W, Ta, Mo, Cr, Re) and the unique single-crystal architecture. Under these conditions, the γ matrix and γ’-strengthening phases remain essentially unaltered, rendering low-temperature annealing insufficient for recrystallization inhabitation [29,36]. Conversely, when the annealing temperature approaches Tm, the effects between elevated thermal activation and residual strain induce drastic microstructural evolution. High-density dislocation networks and defect clusters progressively serve as nucleation sites for the recrystallized grains [21,28,41]. Consequently, conventional high-temperature recovery processes fail to inhibit recrystallization under such thermomechanical coupling.

The rate of migration of HABs during recrystallization is typically linked to the grain growth rate [42], denoted by v, which can be expressed as:

(1) v=MP,

where M represents the grain boundary mobility, and P is the driving force for the boundary migration. The mobility M is typically expressed by the Arrhenius relationship [43]:

(2) $$M=M_{0}e^{-(Q/k_{B}T)},$$

where $M_{0}$ is the pre-exponential factor (a constant), Q is the activation energy, $k_{B}$ is the Boltzmann constant, and T is temperature. From Equation (2), it is evident that the grain boundary mobility increases with the rising temperature.

The driving force *P* for the grain boundary migration is primarily determined by the stored energy resulting from deformation, which is typically related to the dislocation density within the material [44,45]. This driving force can be expressed as:

(3) $$P=\alpha \rho Gb^{2},$$

where α is a constant, ρ is the dislocation density, G is the shear modulus, and b is the Burgers vector. Hence, the driving force is predominantly influenced by the dislocation density within the materials.

From Equations (2) and (3), the grain growth rate is mainly affected by the temperature and dislocation density. Combining Equations (2) and (3), it becomes clear that the grain growth rate is influenced by both the temperature and dislocation density. In general, a lower dislocation density and reduced temperature inhibit recrystallization. However, for nickel-based single-crystal superalloys, the solution process, essential for material homogenization, requires high temperatures. Therefore, it becomes essential to reduce the internal dislocation density through the recovery heat treatment before solution treatment to inhibit recrystallization.

In the case of superalloys, the presence of secondary phase particles (primary γ’ phase in this study) significantly influences grain growth during recrystallization. When the secondary phase particles are fine (smaller than 1 μm), they effectively inhibit the growth of recrystallization nuclei through the Zener pinning effect. The resistance to the grain boundary migration due to these secondary phase particles can be expressed as [46]:

(4) $$P_{z}=\frac{3F_{V}\gamma_{b}}{d},$$

where $P_{z}$ is the Zener pinning force, $F_{V}$ is the volume fraction of the secondary phase particles, $\gamma_{b}$ is the grain boundary energy, and d is the average size of the secondary phase particles. According to Equation (4), the pinning force increases with the volume fraction of secondary phase particles, further hindering the growth of recrystallization nuclei. Therefore, it is crucial to retain a certain proportion of the primary γ’ phase during the recovery heat treatment process to inhibit recrystallization.

In this study, we observed a non-monotonic evolution of GNDs in deformed single crystals at 1000 °C under isothermal heat treatment. Notably, a substantial amount of the primary γ’ phase remained at this temperature. The subsequent recovery heat treatment at 1000 °C for 10 h successfully inhibited recrystallization during the solution treatment process.

## 4. Conclusions

In conclusion, this study explored the temperature-dependent development of the primary γ’ phase content and GND density by annealing tests in a strained third-generation Re-bearing Ni-based single crystal superalloy (WZ30). We successfully discovered an appropriate thermal processing window by studying the association between the annealing temperature and microstructural stability. The isochronal annealing (700 to 1200 °C/1 min) and microstructural investigation demonstrate a key temperature-dependent interaction: At 1000 °C, the GND density decreases non-monotonically, while 62.7% of the main γ’ phase is preserved. Extended 1000 °C/10 h recovery annealing completely suppresses recrystallization during the subsequent 1340 °C solution treatment. This optimized strategy provides a viable solution for improving the microstructural integrity in high-performance turbine components.

## Data Availability

The original contributions presented in this study are included in the article. Further inquiries can be directed to the corresponding author.
